# ﻿A revision of the genus *Cylindroeme* Vives (Coleoptera, Cerambycidae, Cerambycinae)

**DOI:** 10.3897/zookeys.1082.75816

**Published:** 2022-01-20

**Authors:** Yingqi Wang, Guanglin Xie, Wenkai Wang

**Affiliations:** 1 Institute of Entomology, College of Agriculture, Yangtze University, Jingzhou, Hubei, 434025, China; 2 Qingdao Hotincomon Agricultural Materials Marketing Co., Ltd, Jingzhou, China; 3 Hubei Engineering Research Center for Pest Forewarning and Management, Yangtze University, Jingzhou, Hubei, 434025, China

**Keywords:** Longhorned beetles, Oemini, new generic record, new species, Qilian Mountains, China

## Abstract

The genus *Cylindroeme* Vives, 2019 is taxonomically revised. The type species, *Cylindroemevietnamica* Vives, 2019 is redescribed and a second species from Qinghai (China), *Cylindroemeshii***sp. nov.**, is described and illustrated. The genus is redescribed and a key to the known species is presented. The genus is also recorded from China for the first time.

## ﻿Introduction

The tribe Oemini (圆天牛族) currently includes 96 genera (Tavakilian and Chevillotte 2021). Of them, seven were so far known to occur in China (Chen et al. 2019; Tavakilian and Chevillotte 2021).

The genus *Cylindroeme* was established by Vives in 2019; up to now, only the type species, *Cylindroemevietnamica* Vives, described and illustrated from Vietnam, was known (Vives 2019).

In the present paper, the genus *Cylindroeme* is reported from China for the first time with description and illustration of a new species under the name *Cylindroemeshii* sp. nov., based on specimens from Qinghai province. The genus is redescribed and a key to the two known species is presented.

## ﻿Materials and methods

The genitalia were prepared by soaking the whole beetle in boiling water for several minutes, then opening the abdomen from the apex along the dorsopleural margin. The genitalia were then removed with fine forceps and ophthalmic scissors, and later cleared in 10% KOH at 80–100 °C for several minutes.

All habitus photographs were taken with a Canon 5D Mark II digital camera equipped with a Canon EF 100mm f/2.8L IS USM lens and genitalia images were taken with a Leica DFC450 digital camera mounted on a Leica M205A microscope. Images of the genitalia were taken by keeping them in glycerin. All images were edited using Adobe Photoshop 2020.

## ﻿Taxonomy

### ﻿Genus Cylindroeme

Taxon classificationAnimaliaColeopteraCerambycidae

Vives, 2019

5CAC826D-96C8-53FA-B1FD-4927E46A6956

Cylindroeme Vives, 2019: 1. Type species: Cylindroemevietnamica Vives, 2019.

#### Redescription.

Body nearly cylindrical, elongate, equipped with relatively soft integument, clothed with slightly silky pubescence, fringed with sparse hairs on inner side of antennae.

Eyes large, coarsely faceted, deeply emarginate, with large lower lobe. Antennae slender, antennal tubercles feebly prominent and broadly separated, scape thickened apically, remaining segments similar in thickness. Prothorax cylindrical, slightly flat dorsally; pronotum longer than wide, slightly expanded laterally. Scutellum ligulate. Elytra elongate, with subparallel sides; humerus rounded and protruding forward; apex rounded; each elytron with a feeble longitudinal costa on the disc. Prosternal intercoxal process nearly absent, procoxal cavities completely opened posteriorly and strongly angled laterally; mesosternum wide and short, mesosternal intercoxal process rather short and small, triangular, mesocoxal cavities opened posteriorly; metasternum longer than wide, with a longitudinal median sulcus at posterior half; metepisternum rather wide at anterior half and narrowed at posterior half. Abdomen cylindrical, slightly expanded at posterior half, with six (male) or five (female) visible abdomeres. Legs relatively short, femora strongly enlarged, laterally flattened; metatibia about as long as metatarsus, first metatarsomere longer than next two combined.

***Male genitalia*.** Tergite and sternite VIII subrectangular, truncated apically and strongly protruding laterally, with moderately long setae. Tegmen with parameres fused, sparsely clothed with setae apically; ringed part rounded, with lateral arms converged into a long median stem. Median lobe wide and short, slightly arcuate in lateral view, with two long median struts; dorsal apex straight, ventral apex cuspidal. Endophallus thin and long, with two arched basal sclerites.

#### Distribution.

Vietnam, China (new country record).

### ﻿ Cylindroeme vietnamica

Taxon classificationAnimaliaColeopteraCerambycidae

Vives , 2019

8CFA3FD1-2D70-5DE2-9AA9-FDB012E381B5

**Figures 1–8.**
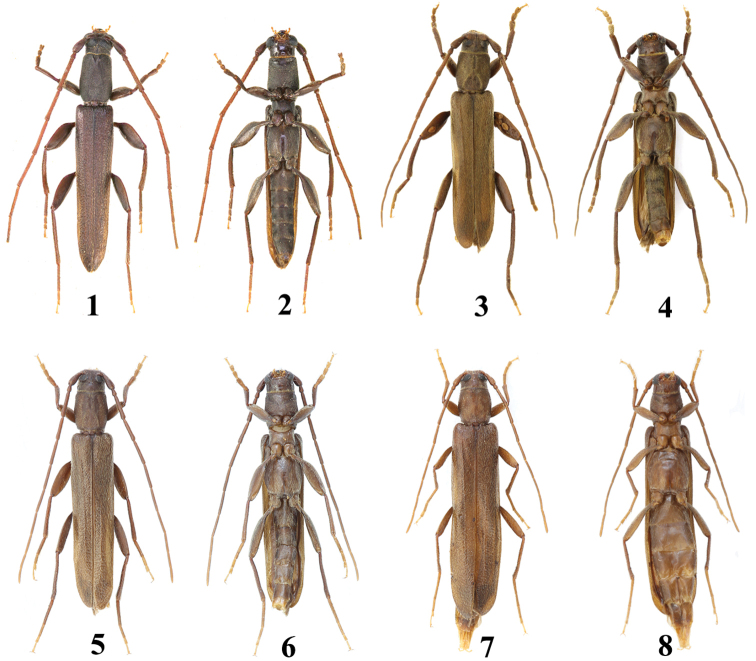
Habitus of *Cylindroeme* spp. **1–2***Cylindroemevietnamica* Vives **3–8***Cylindroemeshii* sp. nov. **1–2, 5–6** holotypes **3–4, 7–8** paratypes **1–6** male **7–8** female.

**Figures 9–21.**
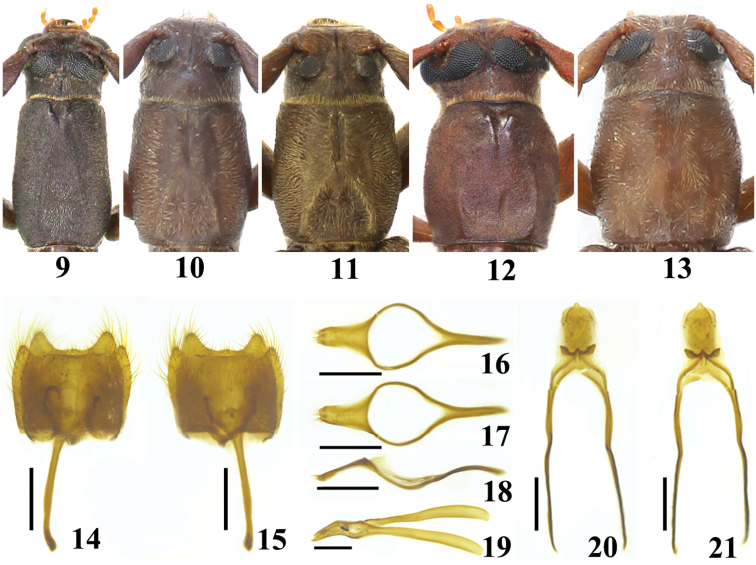
Habitus of *Cylindroeme* spp. **9–13** head and pronotum **14–21** male genitalia **9, 12***Cylindroemevietnamica* Vives **10–11, 13–21***Cylindroemeshii* sp. nov. **9–10** holotypes **11–13** paratypes **9–11** male **12–13** female **14–15** abdominal segments VIII‒IX **16–18** tegmen **19–21** median lobe **14, 16, 20** dorsal view **15, 17, 21** ventral view **18–19** lateral view Scale: 0.5 mm.

扁柱胸天牛 Figs 1–2 , 9 , 12

Cylindroeme vietnamica Vives, 2019: 1–2. Type locality: Lung Cu, Ha Giang, Vietnam. Type depository: coll. E. Vives, Spain.

#### Type material examined.

***Holotype*** (male, Figs 1–2, 9) and one paratype (female, Fig. 12), only examined by photos (provided by Vives).

#### Redescription.

(modified according to the original description and the quality photographs). Male, body length 13 mm, width 2.1 mm (holotype). Body elongate, mostly chestnut-brown, clothed with golden pubescence. Head and pronotum blackish brown, antennae and tarsi brown. Elytra clothed with two types of golden pubescence, one very short and dense, the other relatively long, sparse and arched.

Head parallel-sided behind the eyes; frons trapeziform, with a longitudinal median sulcus; eyes large, strongly emarginate at inner edge; genae rather short. Antennae thin, scape pyriform, strongly punctate, antennomere III clearly longer than antennomere IV, the following segments subequal in length. Pronotum longer than broad, broadest near basal fourth; disc slightly flattened, middle of apical margin equipped with an inverted triangle notch with raised edge, then followed by an inverted V-shaped ridge nearly reaching to posterior edge. Scutellum ligulate, with raised edge. Elytra elongate, about 5.0 times as long as humeral width; humeri rather rounded, protruding forward; sides subparallel, slightly converged towards apex; apex rounded; disc slightly flattened, finely punctate, with a short longitudinal basal rib at the middle of each elytron. Prosternum smooth and shiny on anterior quarter, finely dotted on posterior half; mesosternum transverse, metasternum slightly longer than wide, with a longitudinal median indentation at posterior half, prosternal intercoxal process nearly absent. Abdomen cylindrical, slightly wider at apical half, with six visible ventrites, first ventrite longer than wide, the others shorter and ventrites I–IV with a smooth and shiny posterior border. Legs short, femora strongly widened, laterally flattened, first metatarsomere about 2.0 times as long as next two combined, claws divaricate.

#### Distribution.

Vietnam (Ha Giang province).

### ﻿ Cylindroeme shii sp. nov.

Taxon classificationAnimaliaColeopteraCerambycidae

1A192142-B38E-577C-A472-77BD2915A4CE

http://zoobank.org/E01F5371-2002-4860-90EC-CD2F92A67520

石氏扁柱胸天牛 Figures 3–8 , 10–11 , 13–21 , 22–24

#### Type material.

***Holotype***: male, China: Qinghai, Qilian County, Babao town (八宝镇), Ice Valley (Bing gou, 冰沟), 3110 m, 38°7'60"N, 100°10'21"E, July 25, 2019, leg. Guanglin Xie. Paratypes: 7 males and 1 female, the same as holotype data; 3 males and 3 females, Qinghai, Qilian County, A’rou Township (阿柔乡), Deerhorn Valley (Lujiao Gou, 鹿角沟), 3020 m, 38°8'20"N, 100°24'15"E, July 20, 2019, leg. Guanglin Xie; 5 males, Qinghai, Qilian County, A’rou Township (阿柔乡), Deerhorn Valley (Lujiao Gou, 鹿角沟), 3020 m, 38°8'20"N, 100°24'15"E, July 22, 2019, leg. Guanglin Xie. All of the type specimens are deposited in the Entomological Museum, Yangtze University, Jingzhou, Hubei, China, except for two male paratypes in the private collection of Chen Mou (Shanghai, China) and one male paratype in the private collection of Tianlong He (Anhui, China).

#### Description.

**Male**, body length 8.0–11.0 mm, humeral width 1.5–2.0 mm. Body mostly yellowish brown to light chestnut-brown, clothed with yellowish silky pubescence. Head slightly darker, mandible black apically, clothed with yellowish hairs on outside; labrum and apex of clypeus pale yellow. Antennae sometimes lighter in colour towards to end, fringed with sparse yellowish hairs on inner side of basal segments, more conspicuous on antennomeres III and IV. Pronotum with pubescence obviously silky, forming a squared area on anterior half that looks distinctly different in appearance due to the different direction of the pubescence. Elytra with semirecumbent pubescence. Abdomen with apical ventrite yellowish. Legs with tarsi wholly or only apically yellowish and claw wholly yellowish.

Head about as broad as pronotum, subparallel-sided behind eyes; frons short, subrectangular, with a longitudinal median sulcus; eyes large, upper lobes widely separated; genae very short. Antennae slightly shorter than body, antennomere III slightly longer than antennomere IV, antennomeres V–XI gradually shortened in length. Pronotum longer than broad, broadest behind middle; disc with three narrow longitudinal indentations at apex, median one relatively short, located before the middle, lateral two relatively long, exceeding the middle backwards; with two oblique fine longitudinal ridges at base, anterior ends connected behind the median indentation, forming an inverted V-shaped structure, enclosing a quite flat triangular area. Scutellum ligulate, with distinct edge. Elytra elongate, about 4.2 times as long as humeral width; humeri rounded, slightly protruding forward; sides subparallel; apex rounded; disc finely punctate, with an inconspicuous longitudinal rib at the middle of each elytron, not reaching the apex. Prosternum finely transversely wrinkled apically, mesosternum transverse, metasternum longer than wide, with a longitudinal median indentation at posterior half, pro- and mesosternal intercoxal processes nearly absent. Abdomen cylindrical, distinctly narrower than metathorax, first abdomere obviously longer than others. Legs short, femora strongly widened, laterally flattened, metafemur distinctly exceeding ventrite II, first metatarsomere about 1.4 times as long as next two combined, claws divaricate.

***Male genitalia*.** About apical half of tergite and sternite VIII clothed with sparse setae, longer on sides, apical edge of ventrite VIII more broadly transverse-truncate than tergite VIII. Tegmen waved in lateral view, parameres fused, converged towards apex; apex slightly truncate and sparsely setiferous; ringed part quite rounded, with lateral arms converged into a long median stem; relative length ratios of paramere, vertical diameter of ringed part (except median stem) and median stem equal to 4.84:7.22:6.41. Median lobe wide and short, relative length ratios of median lobe and median struts about 1:3, dorsal apex nearly straight, ventral apex mastoid. Endophallus with two arched basal sclerites connected together.

**Female.** Body length 9.0–10.5 mm, humeral width 1.6–2.0 mm. Similar to male but body colour a little lighter, antennae only reaching behind middle of body, pronotum nearly as long as broad, anterior lateral indentations and posterior longitudinal ridges less obvious than in the male, elytra about 4.3 times as long as humeral width, abdomen with five visible abdomeres, legs slightly shorter, metafemur reaching to about end of second abdomere.

#### Remarks.

The new species can be easily distinguished from the type species, *Cylindroemevietnamica*, by the wider spacing of the upper eye lobes, shorter antennae, less elongate pronotum and elytra, and different discal structure of pronotum.

In the new species, the distance between the upper eye lobes is about as wide as the transverse diameter of a single upper eye lobe (Figs 10, 11, 13), while the upper eye lobes are almost connected in *C.vietnamica* (Figs 9, 12); the male antennomere VIII about reaches to the apical two fifths of the elytra (Figs 3–6), while it nearly extends to the apical one tenth in *C.vietnamica* (Figs 1, 2); the length-width ratio of male pronotum and elytra is about 1.1 and 4.2, respectively (Figs 3–6), while it is about 1.4 and 5.0 in *C.vietnamica* (Figs 1, 2); the pronotum is without a remarkable V-shaped median depression at apex (Figs 10, 11, 13), while it is equipped with a remarkable, broad and short median depression with a V-shaped raised edge in *C.vietnamica* (Figs 9, 12); the male pronotum is equipped with a distinct narrow longitudinal indentation at the apex, extending backwards to the middle and jointed with an inverted V-shaped ridge with two short lateral arms (Figs 10, 11), while the apical median indentation is rather short, inverted triangular, and both lateral arms of the inverted V-shaped ridge are quite long in *C.vietnamica* (Fig. 9).

#### Etymology.

The new species is named after Professor Dr. Fuming Shi (Hebei University, China), a famous Chinese katydid taxonomist.

#### Distribution.

China (Qinghai).

#### Biological notes.

The adults of *Cylindroemeshii* sp. nov. were found on trunks of *Piceacrassifolia* Komarov (Qinghai spruce). They were attracted by half-dead trees whose trunks were bored by xylophagous insects or damaged by other animals. The adults were found crawling and mating on these damaged tree trunks. Based on this, it can be speculated that Qinghai spruce (*Piceacrassifolia*) should be its host plant.

The adults were first discovered on July 20, 2019. At the same time, one egg-laying female adult and one free-moving male adult of *Necydalisinermis* Pu, 1992 (Cerambycidae, Necydalinae) and the free-moving adults of *Tetropiumcastaneum* (Linnaeus, 1758) (Cerambycidae, Spondylidinae) were also found on Qinghai spruce. However, two year later, when the second author visited the same place again on 1–3 August, 2021, no individuals of the new species were found. It indicated that the adults of the new species may be active till the end of July at the latest.

**Figures 22–24.**
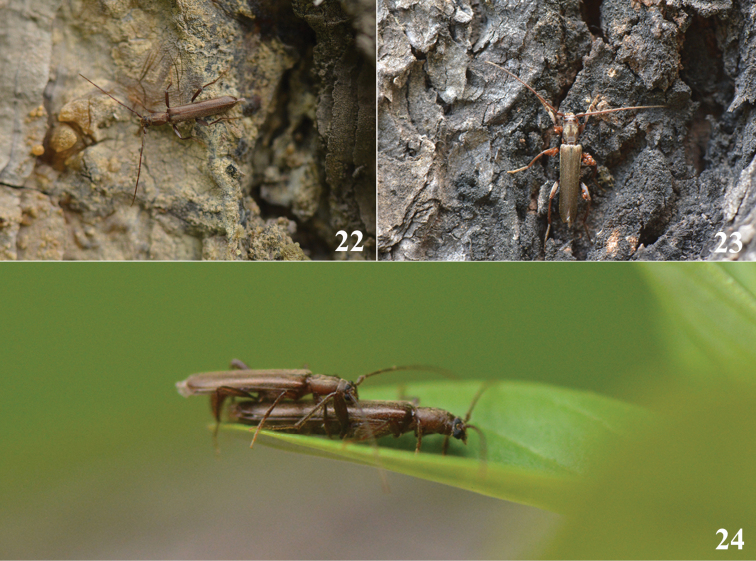
Living adults of *Cylindroemeshii* sp. nov. **22–23** male **24** mating pair, lateral view.

### ﻿Key to the known species of *Cylindroeme* Vives

**Table:** 

1	Upper eye lobes close to each other, almost connected; male with median notch at apex of pronotum rather remarkable, short, inverted triangle shaped, and with inverted V-shaped ridge with lateral arms quite long	***C.vietnamica* Vives**
–	Upper eye lobes widely separated; male with median indentation at apex of pronotum quite long and narrow, and with inverted V-shaped ridge with lateral arms short	***C.shii* sp. nov.**

## Supplementary Material

XML Treatment for Cylindroeme

XML Treatment for Cylindroeme vietnamica

XML Treatment for Cylindroeme shii
